# Temporal and Spatial Synchronicity in West Nile Virus Cases Along the Central Flyway, USA

**DOI:** 10.1029/2022GH000708

**Published:** 2023-05-10

**Authors:** H. M. Hort, M. Ibaraki, F. W. Schwartz

**Affiliations:** ^1^ GSI Environmental Inc. Irvine CA USA; ^2^ School of Earth Sciences The Ohio State University Columbus OH USA

**Keywords:** West Nile virus, Central Flyway, Temporal and Spatial Analysis, Vector Competence, Amplification Factor

## Abstract

This study of West Nile virus (WNV) examined the possibility of avian transmission to explain synchronicity in the year‐to‐year variability of WNV case numbers from Texas northward to the Dakotas, and reasons for the large case numbers on the northern Great Plains. We determined correlation coefficients between annual disease incidence per 100,000 people among states within the Great Plains Region, as well as the Central Flyway. There was spatial and temporal synchronicity, as evidenced by Pearson “*r*,” with values along the core of the Central Flyway (Oklahoma, Kansas, Nebraska, and South Dakota) varying between 0.69 and 0.79. Correlations for North Dakota (*r* = 0.6), however, were affected by local conditions. The concept of relative amplification is helpful in explaining why northerly states along the Central Flyway have larger annual case numbers per 100,000 than Texas but preserve the temporal signal. States differed in their capacity for amplifying the temporal signal in case numbers. For example, Nebraska, South Dakota, and North Dakota case numbers were commonly amplified relative to Texas, with Oklahoma and Kansas deamplified. Relative amplification factors for all states increased as a function of increasing case numbers in Texas. Thus, increased numbers of initially infected birds in Texas likely led to the rapid intensification of the zoonotic cycle as compared to more typical years. The study also confirmed the importance of winter weather in locally modulating disease cases. North Dakota appeared most impacted by these factors to the extent of reducing WNV case numbers in colder years and years with deep snow.

## Key Points

- Spatial and temporal synchronicity in human West Nile virus cases was observed along the Central Flyway
- A consistent pattern of amplification and deamplifications in human case numbers is evident along the Central Flyway as compared to Texas
- Key factors controlling trends include avian spread, vector competence, and local winter conditions, especially in North Dakota

## 1 Introduction

West Nile virus (WNV) is an infectious disease transmitted by mosquitoes. The virus is maintained in an enzootic cycle between mosquitoes as vectors and birds as amplifying hosts. Mammals, such as humans and horses, serve as dead‐end hosts because they do not readily transmit the virus. WNV was first introduced into the United States (U.S.) in 1999 and became endemic across much of North America in just a few years. Despite public attention and efforts with mosquito control programs, WNV has emerged as the leading mosquito‐borne disease in the U.S.

**Figure 1:**
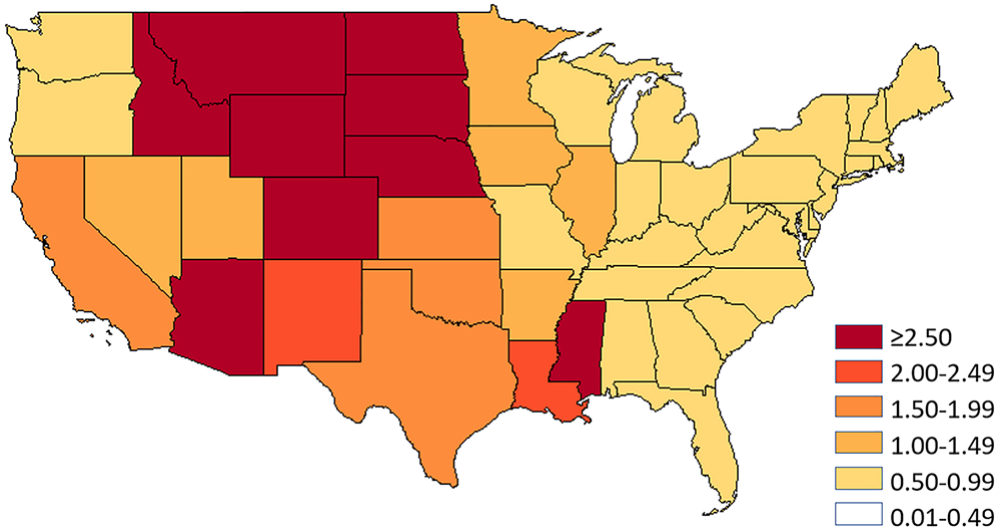
Annual West Nile virus incidence per 100,000 population by state, 1999–2021 (CDC, 2022).

The Centers for Disease Control and Prevention (CDC) operate a nationwide WNV surveillance system (CDC, 2023). It has provided case numbers on WNV human illnesses for nearly two decades. As of 2021, there have been more than 55,000 cases reported, with almost 28,000 as the serious neuro‐invasive form of the disease (CDC, 2023). Examination of this extensive database makes it clear that the numbers of WNV cases in the U.S. have varied significantly both in space and time. Figure 1 shows the average annual incidence rate of total WNV disease cases reported to the CDC by state between 1999 and 2021. Among the 50 states, people living within the Great Plains Region, especially North Dakota, South Dakota, Nebraska, Kansas, Oklahoma, and Texas have been profoundly affected by the virus. The largest WNV outbreaks to date occurred in 2003, which was the year when WNV was introduced to this region.

No vaccines or treatments are currently available for humans, which highlights the importance of better understanding the virus in the hope of reducing annual disease loads. Local‐scale investigations, that is, city or state‐wide, (DeGroote et al., 2008; Gibbs et al., 2006; H. Liu et al., 2008; Mori et al., 2018; Peterson et al., 2008; Ruiz et al., 2010; Winters et al., 2008) have shown that the incidence rates of WNV are impacted significantly by climatic, hydrologic, and socio‐environmental factors. For example, H. Liu et al. (2008) found that the percentage of agricultural land and the total size of wetlands in Indianapolis, Indiana were strongly associated with the numbers of WNV cases. Both agricultural land and wetlands are habitats conducive to mosquito production. Gibbs et al. (2006) found urban‐suburban areas were associated with the occurrence of WNV in Georgia. They concluded that human activities in these settings created water bodies suited to mosquito vectors and bird hosts. A greater opportunity for vector‐host interaction in such settings increased the risk of WNV transmission. These studies suggested that an extremely diverse array of settings or conditions contribute to the complex spatial variability in the number of WNV cases among various locations.

Several comprehensive studies (Bowden et al., 2011; DeGroote & Sugumaran, 2012; Hahn et al., 2015; Paull et al., 2017; Wimberly et al., 2008) have been concerned with environmental associations among the incidence rates of WNV and controlling variables at both national and regional scales. For example, Wimberly et al. (2008) analyzed patterns in WNV occurrence on the northern Great Plains (NGP). They identified a group of counties with a high incidence of WNV, based on climate and land use patterns. They also pointed to a need for finer scales of analyses to understand the relationships between local wetlands and mosquito habitats. In addition, national‐scale studies of WNV dynamics (Bowden et al., 2011; DeGroote et al., 2008; Hahn et al., 2015) were conducted by subdividing counties into sub‐regions to examine regional variability among multiple factors and WNV incidence rates. These studies determined generally that no single variable could be identified that was strongly predictive of case numbers of WNV.

Environmental factors are commonly the focus of studies at local and regional scales with the goal of examining their influence on disease dynamics. However, few studies have investigated the inter‐relationships of disease incidences among multiple regions. One of the processes that could synchronize incidences among multiple states is bird migrations. Several studies have implicated bird migration as a factor in WNV transmission (Hayes et al., 2005; Moon et al., 2019; Swetnam et al., 2018). Swetnam et al. (2018) commented that findings associating isolates of WNV with avian flyways were “not surprising.” Birds are not only the predominant reservoir for WNV, but also important for the transmission of other diseases, such as influenza A and Lyme disease (Swetnam et al., 2018).

Various studies have implicated migratory birds in facilitating the rapid initial spread of WNV (Hadfield et al., 2019; Swetnam et al., 2018). One argument was simply that few other plausible mechanisms were available to explain why the disease could spread so quickly with humans as dead‐end hosts. Since 1999, some 320 species of birds were infected by WNV (Spickler, 2013) with American Robins considered to be super‐spreaders. Various studies supported the idea of long‐distance transmission of WNV with migratory birds along flyways (Moon et al., 2019; Swetnam et al., 2018), which provides a plausible mechanism to explain the spatial correlations in disease cases as well as temporal correlations, which are a focus in this paper.

**Figure 2:**
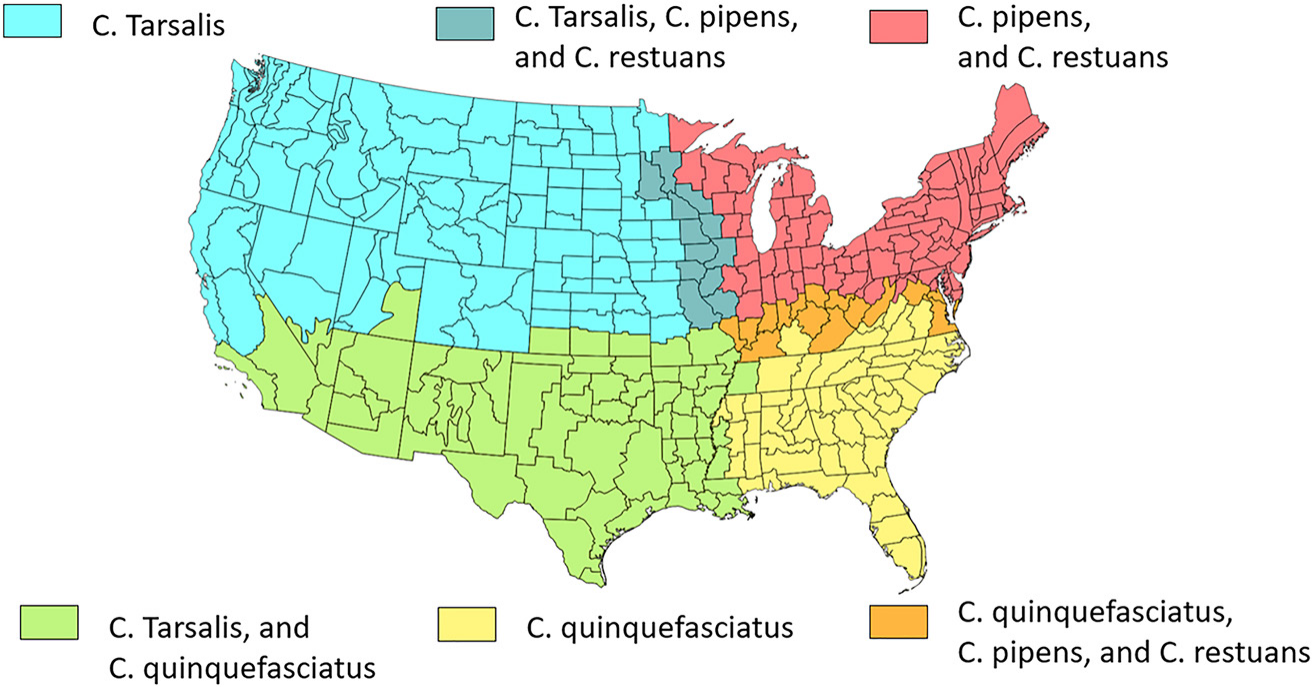
Map showing the spatial distribution of dominant Culex species in the continental U.S. based on Darsie and Ward (2005) (reprinted permission from University Press of Florida), and Data‐driven identification of potential Zika virus vectors by Evans et al. (2017), licensed under CC BY 4.0, Modified from original.

*Culex* mosquitoes are the primary vector responsible for transmitting WNV to humans. The *Culex* genus here in the continental U.S. is comprised of species with varying spatial distributions. The CDC, local public health entities, and vector control districts have been classifying the most predominant *Culex* mosquitoes that transmit the virus in each state. Figure 2 shows the range in the primary *Culex* species based on maps from Darsie and Ward (2005) and Evans et al. (2017). The range of mosquitoes includes six regions based on four *Culex* species.

Here, we hypothesize that spring‐time bird migrations northward from Texas through Oklahoma, Kansas, Nebraska, South Dakota, and North Dakota—the eastern side of the Central Flyway contributed to annual WNV transmission. An avian model for WNV transmission has the potential to explain observed features associated with WNV case numbers and other observations. For example, there are hints of synchronicity in disease expression across the Great Plains Region (DeGroote & Sugumaran, 2012). A year with relatively high case numbers may often be expressed across the Great Plains Region from Texas to North Dakota. Additional support for an avian model came from previous investigations. For example, Swetnam et al. (2018) used phylogeographic approaches to identify North Dakota and South Dakota as sinks for WNV sourced in Texas. Thus, our objective here is to examine the possibility of avian transmission in explaining synchronicity in the year‐to‐year variability of WNV case numbers. A second objective is to elucidate the variability in WNV incidences across the western Great Plains and to interpret causes for variability.

## 2 Methods

### 2.1 Data

#### 2.1.1 Human Disease Cases

The study made use of yearly county‐wide data on total human disease cases from ArboNET Disease Maps (CDC, 2022) between 2003 and 2021. The actual data were embedded in HTML and JavaScript pages, which were processed to provide the needed data. The total human disease cases included both neuroinvasive cases and non‐neuroinvasive cases.

#### 2.1.2 Weather Data

Daily summaries of climate data (1999–2021) were extracted from the Climate Data Online of National Centers for Environmental Information (NOAA National Centers for Environmental Information, 2022) at selected cities in North Dakota, including

- Bismarck Municipal Airport, North Dakota
- Grand Forks University, North Dakota

We made use of weather data, such as the daily average, maximum, and minimum temperature, and snowfall data from station data (NOAA National Centers for Environmental Information, 2022) to correlate with WNV disease incidence on a county‐wide basis. A particular focus of the study was winter weather. Chung et al. (2013) found that winter climate showed a significant influence on case numbers of WNV in Texas. Larger numbers of cases were associated with fewer freeze days. To this end, we calculated heating degree days (HDD) from daily average temperature, and the numbers of freeze days from daily minimum temperature. Heating degree days are the cumulative daily deviation of average temperature above and below 18°C, respectively. The metrics were used as the indicator of cold or warm seasons. Freeze days were counted as those days when the measured average temperature was below −2.2°C, a temperature, which potentially can kill overwintering mosquitoes (Chung et al., 2013).

### 2.2 WNV Disease Incidence Analysis Among States and Along the Central Flyway

The initial analysis investigated the correlation in annual human WNV cases between Texas and all other states in the continental U.S. The choice of Texas as the basis for this comparison was justified because of its southerly position on the Central Flyway and its possible role in controlling disease synchronicity through bird migration. Moreover, Texas had been identified previously as a major source for the circulation of WNV because it, like Illinois, is a point of convergence between the Central Flyway and those to the east (Swetnam et al., 2018).

The study also examined whether correlations exist in annual disease incidence between all pairs of states in the Great Plains Region, that is, Texas, Oklahoma, Kansas, Missouri, Nebraska, Iowa, South Dakota, Minnesota, and North Dakota. For all these correlations, Pearson correlation coefficients (Ott, 1977) and *p*‐values for statistical significance were calculated as a measure of the linear correlation of annual WNV case data between the various pairs of states. We also created a correlation matrix describing the correlation between the pairs of nine states located within the Great Plains Region. This matrix was colourized as a heatmap to help identify highly correlated states. Moran's I spatial autocorrelation (Sugumaran et al., 2009) was not used in this study because we were specifically interested in correlations between Texas and other states within the Great Plains Region.

**Figure 3:**
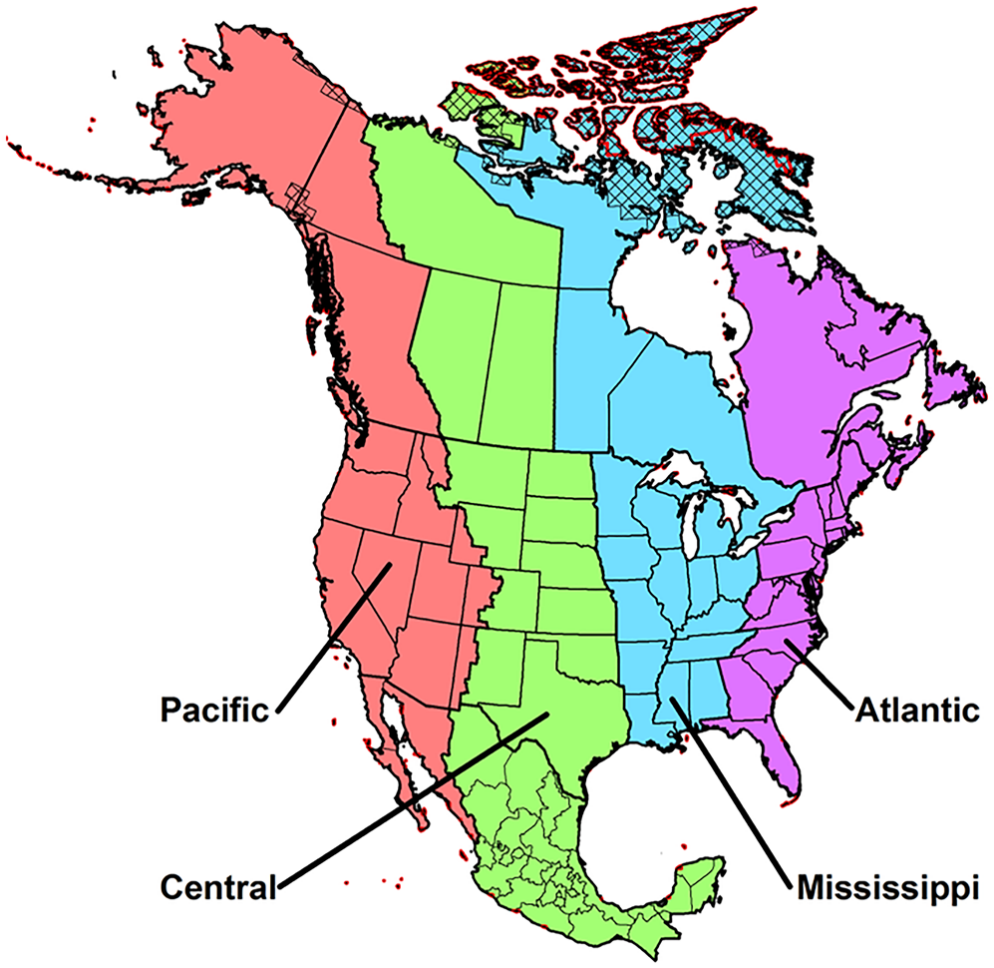
Migratory bird flyways in the U.S (Differential changes in the onset of spring across US National Wildlife Refuges and North American migratory bird flyways by Waller et al. (2018), licensed under CC0 1.0).

A smaller subset of states (Texas, Oklahoma, Kansas, Nebraska, South Dakota, and North Dakota) was chosen to test ideas of WNV dissemination along the Central migratory bird flyway (Figure 3). Our interest was to elucidate the tendency for northerly states (such as North Dakota, South Dakota, and Nebraska) to have higher WNV incidences per 100,000 population, as compared to more southerly states, like Texas (Figure 1). In this analysis, we used the concept of amplification to examine whether there was a discernible expansion in WNV annual case numbers between Texas and other states along the Central Flyway. Amplification involves taking some waveform, preserving its shape but increasing its amplitude or strength. In the context of WNV, mosquitoes interact with birds leading to “amplification,” which in turn leads to the infection of increasing numbers of mosquitoes (Marshall, 2006). With the coming of spring, WNV is amplified within the zoonotic cycle involving mosquitoes and birds. Amplification leads to the infection of increasing numbers of mosquitoes and birds. As dead‐end hosts, human case numbers reflect the amplification of the virus through late spring and summer.

The process of amplification is complicated. Amplification, for example, is likely to be enhanced by the extent of infection in migrating birds (initial loading), vector abundance (Murray et al., 2013), co‐location of birds and mosquitoes due to habitat (Marshall, 2006), drought (Montgomery & Murray, 2015), and other favorable environmental conditions. One important factor in this respect is vector competence, which characterizes the ability of a vector to transmit WNV after being exposed to WNV. As will become clear later in the paper, dominant mosquito species along the Central Flyway differed markedly in terms of vector competence.

In our application, we calculated relative amplification factors using annual disease cases per 100,000 population in flyway states. The basis for comparison was annual case numbers per 100,000 for Texas or

(1) $$A_{Si,TX,yr}=\frac{C_{Si,yr}}{C_{TX,yr}}$$

where *A*_Si,TX,yr_ is the amplification factor for “Si” states along the flyway north of Texas as compared to Texas for a given year, *C*_Si,yr_ is the number of cases per 100,000 in each of the northerly states for a given year, as compared to Texas, *C*_TX,yr_. Thus, a value >1 implies amplification in WNV case numbers relative to Texas. Potential amplification along the Central Flyway lets us quantify the apparent strength of the temporal signal in annual WNV cases, relative to Texas. Although we were interested in situations where the WNV disease signal was strengthened, there were situations where the amplification factor was <1, which is deamplification or weakening of the Texas signal.

### 2.3 WNV Disease Analysis With Climate Factors

Given the experience in Texas with strong associations between winter weather and WNV case numbers (Chung et al., 2013), we examined the potential influence of extremely cold weather in North Dakota. We used Pearson correlation coefficients to examine the strength of relationships that existed between the disease cases and multiple environmental factors, such as the number of freeze days, HDD, and snow depth. The data were cumulatively summed between November and the end of October to compare with the annual total WNV cases.

All the statistical analyses were conducted using Rstudio 202207.1 Build 554 (RStudio Team, 2022) on R version 4.2.0 (R Core Team, 2022). Stats and ggcorrplot (Kassambara, 2019) packages were used and figures are delineated by plotly (Sievert, 2020). Maps were generated based on sp package of R (Bivand et al., 2013; Pebesma & Bivand, 2005) and QGIS version 3.20.1 (QGIS.org, 2022).

## 3 Results

### 3.1 WNV Disease Incidence Analysis Along the Central Flyway

**Figure 4:**
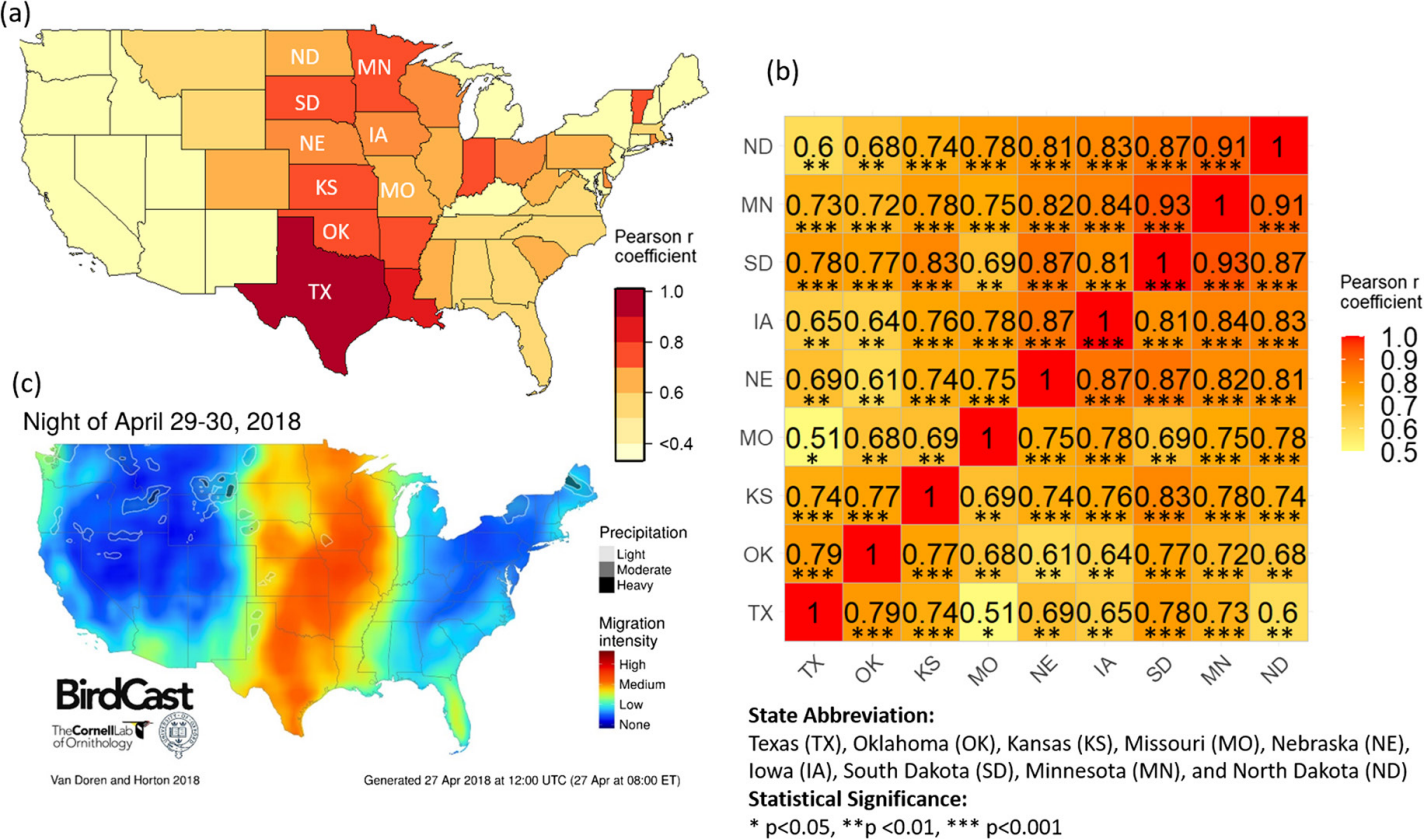
(a) Pearson correlation coefficients created by linear correlations of annual total West Nile virus cases between Texas and each state in the continental U.S., (b) heat map illustrating *r* and *p* values for similar correlations for states in the Great Plains Region, and (c), an example of general migratory bird activity on flyways in the United States (reprinted with permission from BirdCast, Van Doren & Horton, 2018).

With Texas as a focal point, we examined how well the temporal variability in annual WNV case numbers for states was correlated with the Texas numbers. Here, we summarized the values of Pearson coefficients (Pearson's “*r*” values”) from the associated *x*‐*y* plots. Figure 4a is a map of Pearson correlations of annual total WNV cases (2003–2021) between Texas and each state within the continental U.S. The states of the Great Plains Region, along the Central Flyway. Exhibit Pearson's *r* values ≥0.6 (Figure 4a). Correlations also exist for the western portion of the Mississippi Flyway. There were poor correlations with states west of the Rocky Mountains and generally weak correlations with east‐coast states along the Atlantic Flyway (Figure 4a).

Using the same statistical approach, we examined correlations in annual case numbers per 100,000 population for all pairs of nine key states of the Great Plains Region north of Texas. Figure 4b depicts the correlation matrix in annual total WNV cases (2003–2021) between these states. Values closer to 1.0 indicate that two states have a greater correlation. The last row of this matrix shows the correlation of Texas data with each of the flyway states from south to north. The most important trend is spatial and temporal synchronicity of the Pearson correlation coefficients northward along the Central Flyway. This relationship is apparent for most of these states. As will become clear later in the paper, the correlation with North Dakota appeared somewhat weaker, affected by local conditions. Also, correlations with Texas were slightly more robust along the western side of the Central Flyway (Oklahoma, Kansas, Nebraska, South Dakota, and North Dakota) with a mean Pearson *r* of 0.72 as compared to 0.67 for states along the east side of the Mississippi Flyway (Oklahoma, Missouri, Iowa, and Minnesota). Another feature with *r* values in the top right corner of the correlation matrix in Figure 4b is the strong correlation evident among northern states in the Great Plains (i.e., North Dakota, Minnesota, South Dakota, Iowa, and Nebraska). Not only were annual case numbers strongly correlated with neighboring states, but also with other states within this sub‐region. Later in the paper, we explore possible reasons to explain this finding. The existence of temporal and spatial trends in annual case numbers of WNV can be explained by patterns of bird migration along the Central Flyway. In spring, many species of birds surge northward along the Central Flyway. In the Great Plains, migration for some species (e.g., robins) is fostered by warming temperatures and an absence of snow cover. Cold winter storms in the north might delay migration, with birds holding in staging areas further south. Waterfowl returning to wetlands affected by drought in North Dakota could simply keep moving north into Canada. These local complexities could impact the development of WNV in the upcoming warm season.

Figure 4c is an illustrative example showing the behavior of birds in the early spring of 2018 (Van Doren & Horton, 2018). This graphic based on weather radar shows how migratory birds were concentrated along flyways through the Great Plains Region. On the night of 29 and 30 April 2018, there was high migration intensity along the entirety of the Central Flyway and parts of the Mississippi Flyway. A qualitative comparison of Figures 1 and 4c, shows similar trends with WNV cases and migratory pathways through the Central Great Plains. Beyond our correlation analyses of annual WNV case numbers, this result is also supported by the phylogeographic work (Swetnam et al., 2018), which discussed how migratory birds disseminated the virus along flyways crossing the Great Plain from south to north in early spring.

To provide additional perspectives on these results, we present examples of several of the plots upon which the correlation matrix (Figure 4b) is based. Figure 4a shows the correlation of yearly WNV disease cases between North Dakota and Texas from 2003 to 2021. The data were log‐transformed, and the straight line is the linear regression model. The regression line uses the annual WNV disease cases in North Dakota and Texas as the dependent and independent variables, respectively. The Pearson correlation was 0.60 and highly significant (*p* < 0.001), with a large deviation indicated for 2009. This deviation is sufficiently large to affect the Pearson *r* for the plot. If the data point for 2009 was not considered in the present regression analysis, the *r* value would have increased to 0.65 (*p* < 0.001). We mention this point because, as will become clear, 2009 was a winter‐weather outlier—cold with little snow.

**Figure 5:**
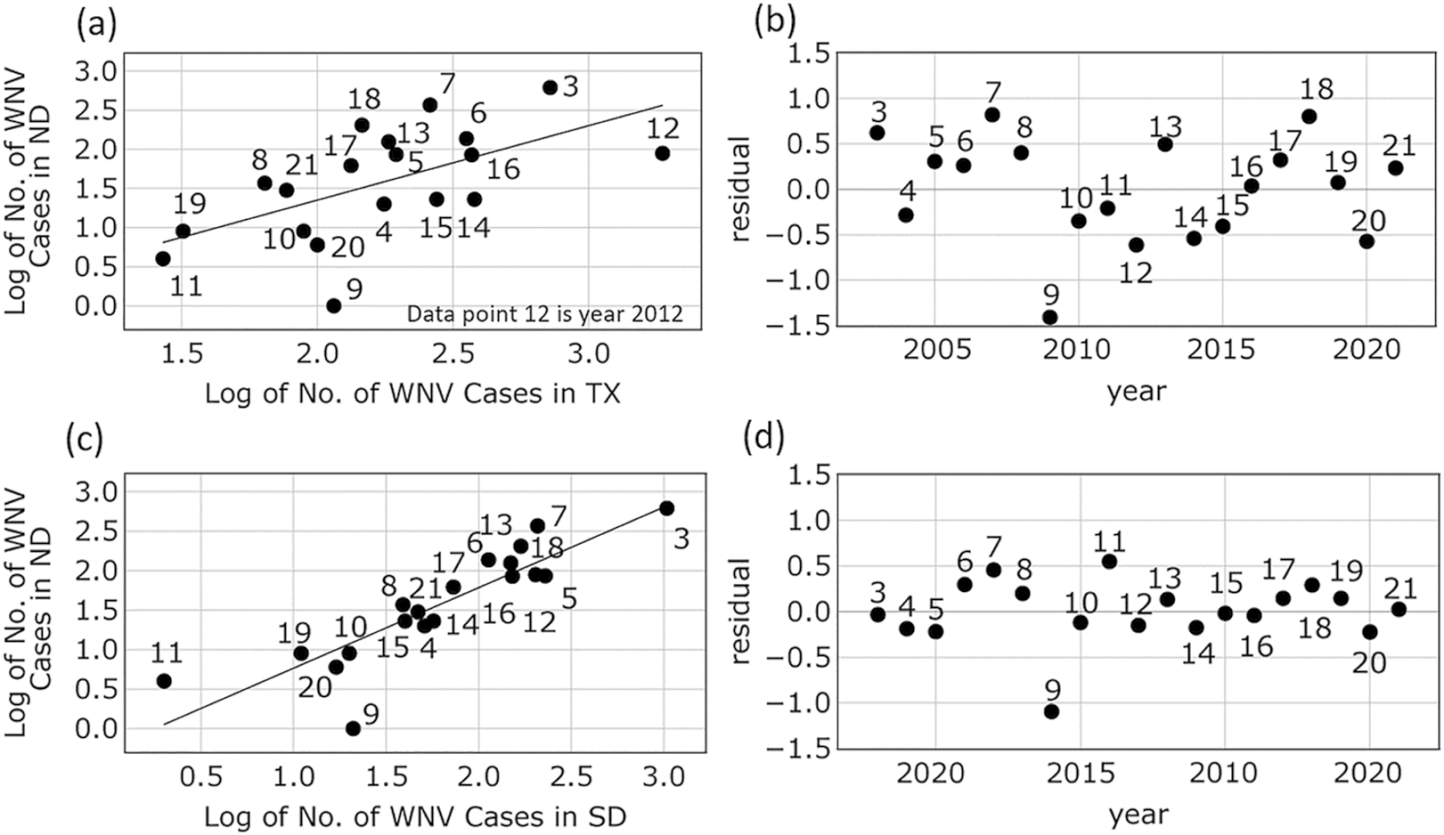
Statewide correlation analysis. Panels (a, b) show linear regressions between North Dakota (ND) and Texas (TX) and residual deviates from the linear line for each year, respectively. Panels (c, d) present the linear regressions between ND and South Dakota (SD), and residual deviates from the linear line for each year, respectively.

Figure 5b shows the residual which is the difference between the observed WNV disease cases in North Dakota and the fitted value based on the linear regression line. Any years above 0 means that the WNV disease cases for North Dakota were smaller than expected, based on estimated values from the linear regression model. A value less than 0 means the disease cases were higher in North Dakota than expected from WNV cases in Texas.

The correlation in annual case numbers was plotted for South Dakota versus North Dakota (Figure 5c). The Pearson correlation coefficient was 0.87 (*p* < 0.001) for these adjacent states. For 2009, potentially associated with the winter‐weather deviations noted in Figure 5a, it is also associated with a deviation in the South Dakota/North Dakota comparison (Figure 5c). This result suggests that the reduction in total case numbers only occurred in North Dakota.

**Figure 6:**
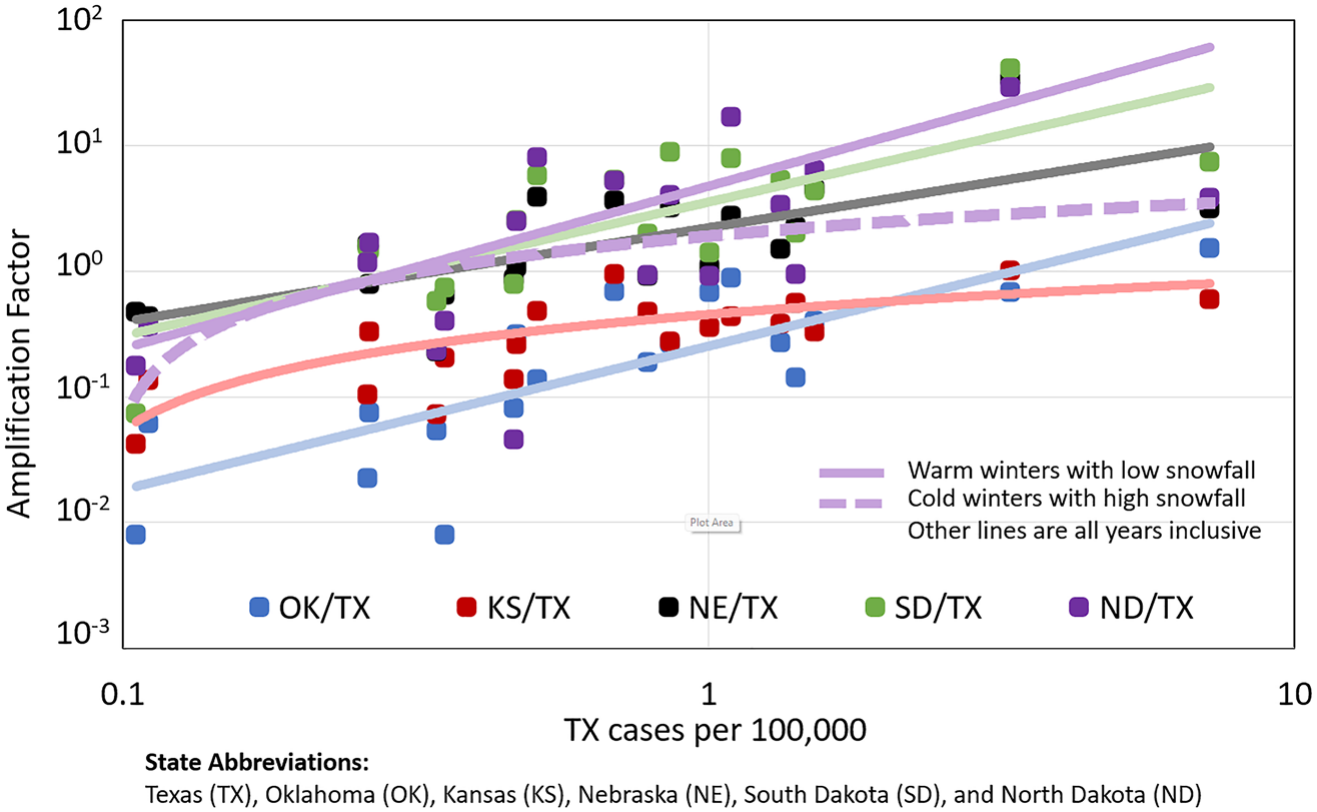
The amplification factor between Texas and each state along the Central Flyway changed as a function of disease cases per 100,000 population in Texas. The two lines for North Dakota/Texas reflected variability in winter weather in North Dakota. Data for all years are plotted for the indicated states. Trend lines were generated using logarithmic and power fittings.

The remainder of this section uses the concept of relative amplification in annual case numbers to explain the tendency for more northerly states along the Central Flyway to have larger annual case numbers per 100,000 population than Texas. Questions that we considered were (a) were there obvious factors that influenced these case numbers and (b) were there spatial patterns in disease amplification moving northward from Texas → Oklahoma, Kansas, Nebraska, South Dakota, and North Dakota. Figure 6 compares the amplification factors calculated using annual case numbers per 100,000 population for those states as compared to Texas (Equation 1). Amplification factors for pairs of states (i.e., Oklahoma/Texas, Kansas/Texas, etc.) were not constant but commonly varied over several orders of magnitude. Moreover, for all pairs tested, amplification varied as a function of the Texas cases per 100,000 population (Figure 6). In years where the case numbers per 100,000 in Texas were relatively small, they were also minimal in the other states. In other words, amplification factors were commonly <1. Years with relatively larger case numbers in Texas were associated with amplification of case numbers in Nebraska, South Dakota, and North Dakota. Thus, case numbers in some years were larger than Texas. Thus, the five states north of Texas have a time series signal like Texas but with strengths (i.e., amplitudes), which are stronger or weaker depending upon the relative intensity of WNV disease cases.

Lines fitted through the amplification/deamplification factors (Figure 6) define a set of unique amplification functions for Oklahoma, Kansas, Nebraska, South Dakota, and North Dakota. These functions have a similar shape but are stacked with Oklahoma and Kansas toward the bottom, Nebraska in the middle, and South Dakota and North Dakota toward the top. The data are obviously noisy but suggest that significant amplification of WNV case numbers per 100,000 population occurred commonly with North Dakota and South Dakota, sometimes with Nebraska when case numbers in Texas were relatively high, and rarely for Oklahoma, and Kansas. In Figure 6, the data for North Dakota appear scattered to the extent, for example, that 1 case per 100,000 in Texas could result in either relatively smaller or larger amplification factors. Thus, for North Dakota, there are two amplification lines plotted (Figure 6). The following section examines additional, unaccounted factors for North Dakota that can reduce the correlations with Texas.

### 3.2 WNV Disease Analysis With Climate Factors

Our previous investigations in North Dakota (Mori et al., 2018) found that variability in summer weather by itself was unsatisfactory in explaining the variability in WNV case numbers per 100,000 population. Moreover, papers by Chung et al. (2013) and Wimberly et al. (2014) motivated us to investigate winter weather conditions and how they might have influenced patterns of amplification for North Dakota. As a measure of cold from November to October, we took measured daily temperatures and converted them to HDD, and the number of freeze days. Burleigh County in central North Dakota was selected because it was always among the areas most affected by WNV. We also utilized total snowfall data from the airport weather station in Burleigh County, North Dakota. The annual total of WNV disease cases and weather variables were used for this comparison.

**Figure 7:**
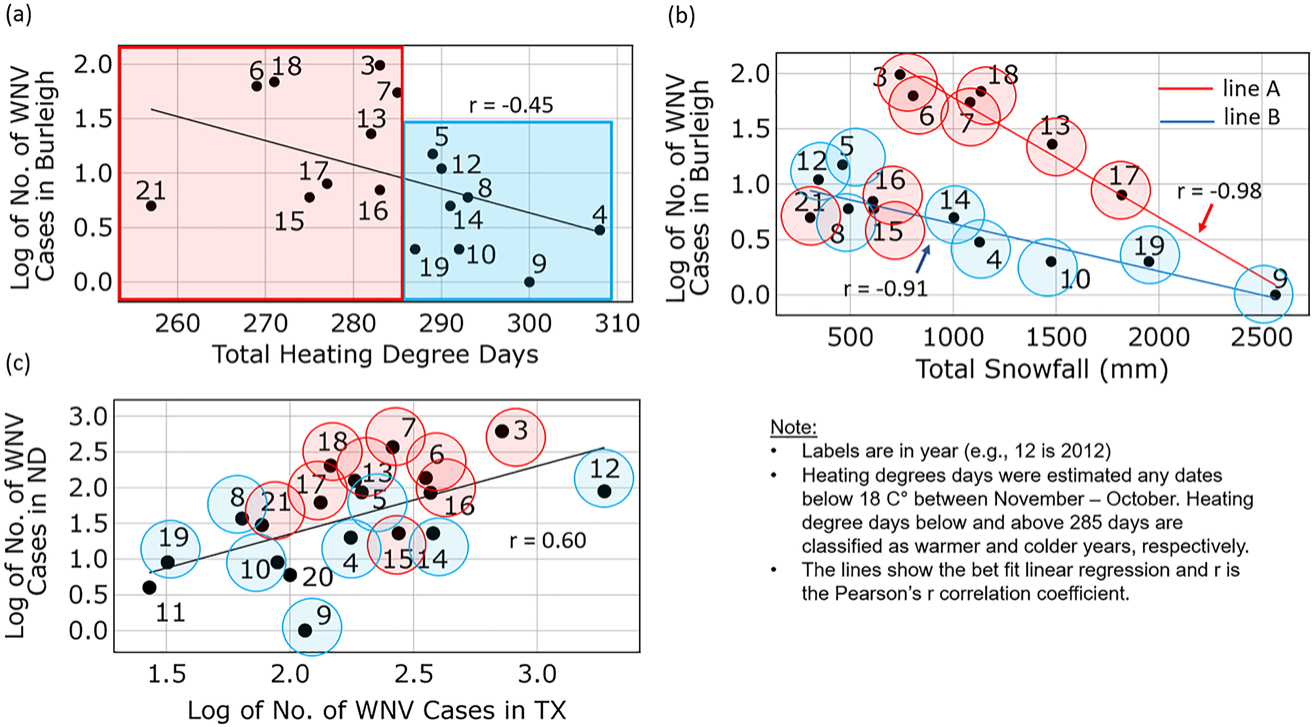
Panel (a) shows a weak correlation between case numbers and heating degree days (HDD). The HDD are divided into a warm‐weather set (red) and a cold set (blue) for reference purposes. Panel (b) shows the correlation between total snowfall and annual disease cases described by two lines. The top line characterized by relatively low HDD represents warmer conditions. The lower line represents colder conditions. Panel (c) shows the correlation between the annual West Nile virus cases in Texas and North Dakota with smaller case numbers in colder years and larger case numbers in warmer years.

Figure 7a is a logarithmic plot of total case numbers versus total HDD for Burleigh County, where Bismarck, the capital city of North Dakota is located. The year 2004 had the highest number of total HDD while 2021 had the lowest. Despite the extreme weather, the disease deviations from the mean were small (Figures 5b and 5d) between North Dakota and Texas, and between North Dakota and South Dakota. The numbers of HDD are weakly, negatively correlated with WNV case numbers. The indication is that WNV case numbers in Burleigh County tended to be low when the weather was colder. The weak correlation with HDD suggests, however, that other unaccounted factors exist. To this end, we examined the potential role of both total HDD and total snowfall (from November to the following October).

Total snow depth emerged as a second important variable in helping to explain variability in disease cases. Figure 7b shows the correlation between total snowfall from the City of Bismarck and log‐transformed disease cases for Burleigh County. This figure clearly shows two distinct correlations (lines A line B). Pearson correlation coefficient showed that the total snowfall and disease cases were highly negatively correlated for both lines (line A: *r* = −0.98; *p* < 0.001, line B: *r* = −0.91; *p* < 0.001). In other words, years with larger snowfalls during November—April, before the start of the mosquito season, were associated with relatively smaller WNV case numbers.

The two lines place snowfall in a temperature context. For example, years with smaller numbers of HDD (i.e., warmer winter weather) are plotted along line A. Colder years with larger values of HDD clustered mostly along line B. Thus, in Burleigh County, the largest case numbers were associated with low total snowfall amounts and warm winter weather. The smallest number of cases were associated with cold winters and relatively large snowfall amounts. Although these relationships are not without exceptions, results suggest that the combination of HDD and the total snowfall might provide an early warning of likely summer WNV cases.

In an earlier section, we suggested that the relative amplification of Texas case numbers in North Dakota might be impacted by winter weather. In other words, local factors have produced variability in the expected pattern of amplification. Figure 7c shows the correlation between total disease cases in Texas and North Dakota. As is evident visually, the best‐fit line through all the data yielded a weak correlation with an *r*‐value of 0.60. However, the coloration of the dots shows how, for example, colder winter weather indicated by HDD created fewer than expected WNV cases, resulting variability that effectively reduced the strength of the correlation. This finding also suggests that local factors (snowfall and HDD) have added additional complexity to the regional correlation between North Dakota and Texas.

## 4 Discussion

Our study identified a “Midwest signal” in WNV cases along the Central Flyway. Given the dynamics of spring‐time bird migration along the Central Flyway, case data for Texas provides the logical basis for comparison. For example, Roehr (2012) referred to Texas as “the epicenter of an outbreak of WNV in the United States.” Swetnam et al. (2018) identified a south‐to‐north pathway for WNV from Texas north into South Dakota.

Annual case numbers per 100,000 population of Texas were correlated with Oklahoma, Kansas, Nebraska, and South Dakota with *r* values falling in a range from 0.69 to 0.79 (Figure 4b). These results pointed to spatial and temporal coherence with data from Texas. For North Dakota, however, the correlation with Texas was weak and departed from this pattern. We presented evidence that implicated local, cold weather as an additional factor impacting case numbers.

We propose that some proportion of migratory birds staging in Texas are carriers of WNV. The northward migration of those birds seeds the zoonotic bird/mosquito cycle as the overwintering mosquitoes emerge. The size of that proportion is variable depending on conditions in Texas. For example, more freeze days in Texas appear to reduce WNV impacts with fewer human case numbers (Chung et al., 2013). Drought conditions there in 2012 have been implicated for the massive outbreak of WNV in 2012 (Roehr, 2012).

The total human case numbers for a state reflected the extent to which viral amplification occurred through late spring and summer. For states closest to Texas along the Central Flyway, that is, Oklahoma and Kansas, deamplification of the Texas case numbers means that although case numbers per 100,000 are correlated with Texas through years (*r* values 0.79 and 0.74, respectively), the strength (i.e., magnitude) of the temporal signal was weaker. In effect, for most years, infections, and the basic reproduction number remained low. The reasons for this deamplification are unclear and could relate to relatively short bird residence times during migrations with limited co‐mingling, unfavorable habitats for mosquitoes, or poor vector competence that attenuated spread.

As shown in Figure 6, in these states and all others, the amplification factors increased as a function of increasing case numbers per 100,000 population in Texas. Thus, when WNV case numbers are relatively high in Texas, the amplification factors for Kansas and Oklahoma approach one and case numbers per 100,000 will be comparable to Texas (Figure 6). We suggest that larger numbers of infected birds magnified the early numbers of zoonotic infections ultimately leading to larger annual human case numbers.

Moving northward to Nebraska, there was evidence of actual amplification in human cases relative to Texas, once case numbers for Texas exceeded about 0.5 cases per 100,000 (Figure 6). A maximum amplitude value of approximately 6 was possible when cases in Texas reach approximately 2 cases per 100,000. For South Dakota, amplification factors were commonly around 5 with indications of values as high as 42. The potential exists for significant amplification in North Dakota for certain years when case numbers in Texas are the worst. Yet, in some years, the amplification can be less than Nebraska. We attribute the variability in North Dakota to extreme local winter conditions. For example, the years with the four highest amplification factors (upper purple line, Figure 6) were 2003, 2006, 2007, and 2018. All these years were warm with low snowfalls which are represented by line A in Figure 7b. In addition, the years with the four lowest amplification factors (lower purple line) were 2009, 2011, and 2020. The year 2009 was a cold year with a high snowfall. The other two years had no record of WNV but 2011 and 2020 were high snowfall years with relatively high numbers of freeze days (Figure S1 in Supporting Information S1).

Several processes and factors likely contributed to the change in the pattern of development of WNV cases along the Central Flyway from Nebraska northward. One possible factor in this respect is vector competence, which characterizes the ability of a vector to acquire and transmit WNV. Given a mosquito population of some given size, a population of competent mosquito species will amplify the initial spring‐time signal and grow a WNV infection more quickly as compared to a less competent population.

Table 1 summarizes data on vector competence for *Culex* species commonly found in the U.S. The method for determining competency involved calculating the proportion of WNV infected mosquitoes with positive salivary secretions (Dodson et al., 2012; Ebel et al., 2005; Goddard et al., 2002), and the recovery of WNV from birds' blood, which were fed with infected mosquitoes (Sardelis et al., 2001; Turell et al., 2001). Indications are that *Culex tarsalis* is the most virulent species followed by *Culex restuans*, *Culex pipens*, and *Culex quinquefasciatus* (Table 1). This finding is consistent with Turell et al. (2005).

**Table 1:** Summary of Vector Competence for Four Culex Species

*Culex* species	Vector competence (%)	Location	Reference
*Culex quinquefasciatus*	0–20	TX	Sardelis et al. (2001)
	0	CA	Goddard et al. (2002)
*Culex pipens*	2.6	NY	Ebel et al. (2005)
	20	NY	Turell et al. (2001)
*Culex restuans*	4.7	NY	Ebel et al. (2005)
	40.3–42.5	IL	Mutebi et al. (2012)
*Culex tarsalis*	82	CA	Goddard et al. (2002)
	51–78	CA	Dodson et al. (2012)

Of the *Culex species* shown in Figure 2, *Culex tarsalis* is commonly present in states along the Western Flyway. Yet, it is not always the dominant species. For example, in Texas, *Culex quinquefasciatus* is more abundant than *Culex tarsalis* (Molaei et al., 2007; Morin & Comrie, 2013); *Culex pipens* is far more dominant than *Culex restuans* in Illinois (Hamer et al., 2008; Ruiz et al., 2010). The geographic information from Figure 2 and Table 1 suggests that there will be potential changes in the behavior of WNV along the Central Flyway, as a function of the relative abundance of the extremely competent *Culex tarsalis*. Expectations are that disease incidence would be higher in the NGP Region compared to eastern and southern states because *Culex tarsalis*, the most competent vector species is the dominant *Culex* species in the area.

Another factor leading to large amplification of human case numbers in North Dakota and South Dakota is localized occurrence of wetlands and pothole lakes that co‐locates breeding sites for mosquitoes and certain bird species. The Prairie Pothole region located in the NGP is unique in the world with respect to the sheer numbers of small ponds and wetlands (G. Liu & Schwartz, 2011).

One of the local factors that we identified for North Dakota is snowfall. More snowy years lead to fewer WNV infections. Often, snow cover will delay the arrival of migrating birds in early spring because of inaccessible food sources (Marra et al., 2005). Snowmelt runoff water flowing into potholes in the early spring does not likely produce an optimal mosquito habitat because of changing water levels and cold water‐temperatures. Therefore, those years with abundant snowfall will minimize the WNV transmission between birds and mosquitoes and delay the growth of mosquito populations to later in the season. Warmer winters appear to benefit mosquitoes. For example, in Burleigh, North Dakota, WNV case numbers were somewhat correlated to years with warmer winters as indicated by HDD (Figure 7a). More work is needed to establish whether cold‐related deaths of overwintering mosquitoes would be an important factor here in North Dakota, leading to increased WNV cases. Chung et al. (2013) and Wimberly et al. (2014) suggested that a warmer year makes it easy for mosquitoes to overwinter and enhance the early start of the spring which may amplify the spread of WNV between mosquitoes and birds (Marini et al., 2021; Shaman et al., 2011).

Neither winter temperatures, reflected by HDD nor snowfall amounts by themselves were able to explain case numbers as WNV for North Dakota. Results in Figure 7, provide preliminary evidence that these parameters taken together might provide a basis for predicting WNV in North Dakota in subsequent summers. Unlike the other states along the flyway, the extreme winter weather that sometimes occurs in North Dakota appears to perturb correlations in human WNV cases along the flyway.

There are limitations in comparing total HDD at Bismarck Municipal Airport, with state‐wide total disease cases. Thus, there are spatial scaling issues inherent in comparisons in Figures 7b and 7c. However, arguments can be made for using dynamics of WNV incidence in Burleigh County as a proxy for the total WNV cases in North Dakota. Twenty percent of disease cases in North Dakota occurred in Burleigh County and case numbers are highly correlated with the total number of disease cases in North Dakota (*r* = 0.93, *p* < 0.001, Figure S2 in Supporting Information S1). Additionally, while snowfall amounts were different, the relative trends in annual snowfall were similar to other cities in North Dakota (*r* = 0.69; *p* < 0.001) as compared to Grand Forks, North Dakota (Figure S3 in Supporting Information S1). Therefore, we presume that the comparison we made between Figures 7b and 7c is generalizable.

It was generally beyond the scope of this investigation to explain the variability in case numbers for Texas. It appears that WNV in Texas is influenced by a complex array of climatic and social factors, along with physiological factors affecting birds and mosquitoes (Montgomery & Murray, 2015; Wimberly et al., 2014). Winter weather expressed as freezing degree days for Dallas County showed a negative correlation with the number of WNV cases (Chung et al., 2013). Roehr (2012) reported that the massive WNV outbreak in Texas could be related to drought and increased bird numbers.

### 4.1 Statistical Considerations

Our analyses relied on linear and non‐linear correlations of data. There are well‐known issues around such correlations, which influence the confidence in interpretations. The first relates to sample size (*n*). The regression analyses of annual case numbers involved 19 years of annual data, which is small and less than optimum. However, these were not designed experiments and 19 years represented the totality of available data. We conducted a sensitivity analysis to evaluate how the length of the time series in case numbers might affect the state‐to‐state correlations (Figure S4 in Supporting Information S1). It showed that short time series would result in correlations with spuriously high Pearson *r* values (Figure S4 in Supporting Information S1). Once values of *n* exceeded about 10 the curves exhibited asymptotic behavior. With values of *n* approaching the maximum 19, Pearson *r* values appeared to converge with some oscillation evident (approximately ±0.03). The complete analysis, however, involved 90 independent regression analyses, given the paired analyses of nine different states (Figure 4b). Altogether, the outcome of correlations was consistent and significant with reasonable Pearson correlation coefficients. The main limitation with small “*n*” correlations is that they are limited to identifying “big” effects (Sauro, 2013). The WNV human case data, spread relatively uniformly across four orders of magnitude, represent a big effect.

Moving to the analyses of amplification factors versus Texas case numbers (Figure 6) these correlations were inherently noisy, which we acknowledged, and in our opinion did not warrant rigorous, statistical fitting. The illustrative lines show consistencies in the nonlinear shapes of the various curves and a logical pattern of ordering with the regression lines. The analyses of cold weather impacts for North Dakota with Figure 7b involved regressions with a very small *n*. Another limitation is the warmer and colder years did not segregate cleanly as indicated by colored data points (e.g., Figures 7b and 7c). These preliminary results for North Dakota, however, were intriguing and potentially useful in explaining why North Dakota/Texas comparisons in case numbers, and magnification factors behaved differently than others. Our description recognized the preliminary character of these winter results for North Dakota.

A second issue with regression is that correlation does not necessarily imply causation. Definitive elaboration of causation normally requires well controlled experiments, for example, as occurs with pharmaceuticals. The data for this study were obviously not the outcome of a controlled experiment. Here, we describe how knowledge and contextual information can help in establishing causality (Hill, 1965). There are nine criteria useful in inferring causation (Fedak et al., 2015; Hill, 1965).

We think inferences with respect to WNV case correlations, and amplification factors versus Texas case numbers have met several of Hill's nine criteria for demonstrating causality. His list included strength of association, consistency, specificity, temporality, biological gradient, plausibility, coherence, experiments, and analogy. For example, we will elaborate on the implications with respect to three criteria. Plausibility and coherence are similar in their examination of the question as to whether associations make sense considering existing knowledge. Our analyses acknowledge that the early spread of WNV away from the east coast of the United States was due to “highly mobile avian reservoirs” (Swetnam et al., 2018) and migratory birds more generally (Peterson et al., 2003). Our correlation results among pairs of states were in line with ideas from Swetnam et al. (2018), discussed previously. They used phylogenetic analyses (a) to identify a south to north transmission pathway from Texas to North Dakota and South Dakota and (b) to elucidate the important role of Texas in the circulation of WNV. Coherence with flyway‐based conclusions of Moon et al. (2019) for the circulation of WNV also added plausibility.

Data from the surveillance of dead birds were somewhat helpful in contributing to plausibility. Such studies were emphasized early in WNV studies (Petersen, 2019). Although initially helpful in providing an early warning of the potential human WNV risks, they were phased out for many reasons (Petersen, 2019). Such studies, however, were important in demonstrating the capacity of migratory birds to spread WNV. For example, Sullivan et al. (2006) conducted a serological survey of harvested red‐winged blackbirds from North Dakota's migratory population in 2003–2004. The study indicated that as many as 2 million birds may have been infected in North Dakota with important implications for the southward fall migration, and for the potential of the very large population of red‐winged blackbirds to serve important WNV spreaders within the U.S.

Across the Great Plains, WNV was endemic, reoccurring each year. In Texas, WNV was active all year along the Gulf Coast of Texas and Louisiana, as indicated by analyses of dead birds and adult mosquitoes (Tesh et al., 2004). However, during the cooler months, virus activity was at a low level. In colder parts of the Great Plains (e.g., North Dakota and South Dakota), there is no virus activity in winter. Mosquitoes enter diapause in fall and carry the virus through the winter (Wimberly et al., 2014). Our correlations of winter‐weather impacts on case numbers are plausible based on previously mentioned studies (Chung et al., 2013; Wimberly et al., 2014). We noted similar cold weather impacts for North Dakota. While severe cold will increase the mortality of overwintering mosquitoes, the combination of snow and cold in North Dakota, can also impact the timing of bird migrations northward, thereby impacting the early stages of viral amplification. Although, correlations with Texas case numbers and amplifications in cases were reduced in years with extreme winter weather, the correlations between Texas and North Dakota remained. This result suggests warm weather endemicity is related to avian transfer along the flyway.

The last of Hill's (1965) criteria useful in establishing causality is analogy. There are comparable studies with an accepted causal relationship that could inform our work (e.g., Fourment et al., 2017). Avian influenza viruses, such as H5N1, infect migratory birds, such as ducks and geese that travel long migration routes. Phylogeograhic studies found a north‐south pattern of disease spread along known North American flyways. Migration rates for avian influenza viruses tend to be higher within flyways than across flyways and point to an association between viral dispersal and bird migration routes.

## 5 Conclusions

This study was designed to elucidate coherences in temporal and spatial scales of transmission patterns for WNV. We described correlation in human case numbers between Texas and states along the Central Flyway to North Dakota and South Dakota. These findings reinforced previous indications from genetic‐based studies of WNV dispersal along migratory bird routes and the unique role of Texas as a source for WNV spread northward (Swetnam et al., 2018).

The annual fluctuations in WNV cases in Texas were translated northward, providing coherence temporally to states along the Central Flyway. However, states differed in their capacity to enhance the temporal signal. In Oklahoma and Kansas, deamplification in case numbers relative to Texas was most often observed except for the worst WNV years for Texas. For Nebraska, South Dakota, and North Dakota case numbers were commonly amplified relative to Texas. Relative amplification factors in all states increased as a function of increasing case numbers per 100,000 in Texas. Increased numbers of initially infected birds likely led to the rapid intensification of the zoonotic cycle and virus spread, as compared to more typical years.

The factors contributing to deamplification in case numbers for Oklahoma and Kansas are uncertain. Possible factors include shorter residence times for migratory birds, less than optimum habitat for birds and mosquitoes, or poor vector competency. Amplification of cases in Nebraska, South Dakota, and North Dakota coincided with the transition to *Culex tarsalis* as the dominant mosquito vector. This species is by far the most competent *Culex* vector as compared, for example, to *Culex quinquefasciatus* which dominates in areas south of Nebraska. Another relevant factor for South Dakota and North Dakota is likely the pond wetland habitats of the Prairie Pothole region which leads to the co‐location of mosquitoes and migratory birds.

Our study provides additional evidence supporting the importance of winter weather. It is likely that large snow accumulations modulate the springtime return of migratory birds. North Dakota appeared most impacted by these factors to the extent of reducing WNV case numbers in colder years and years with deep snow. Such correlations between the local WNV disease cases and these factors may provide a robust, early screening tool for predicting the severity of WNV cases for the following summer in North Dakota. These local factors at work in North Dakota tend to reduce the correlation evident with case numbers in Texas.

The magnitude of WNV infections in Texas sets the stage for infections for states along the Central Flyway in springtime. Our work supports the concept of a Texas source for WNV with sinks in North Dakota and South Dakota, as determined through phylogeographic analyses (Swetnam et al., 2018). A focus on WNV surveillance in Texas, as suggested by Swetnam et al. (2018), is also warranted because of the possibility of summer disease predictions for more northern states.

Our findings here are important because they provide a framework for evaluating the impact of local conditions within a regional context. Future studies will examine other patterns of dissemination out of Texas.

## Conflict of Interest

The authors declare no conflicts of interest relevant to this study.

## Supporting information

Supporting Information S1

## Data Availability

Data from the ArboNET Disease Maps (CDC, 2022), which is an arbovirus surveillance system and Find a Station (NOAA National Centers for Environmental Information, 2022), which is a data tool for weather record were used for the basis of this manuscript. Data from both these sources are freely available. The list of weather station IDs and station names we used in the analysis are follows. USW00024011: BISMARCK MUNICIPAL AIRPORT, ND US (https://www.ncdc.noaa.gov/cdo-web/datasets/GHCND/stations/GHCND:USW00024011/detail). USC00323621: GRAND FORKS UNIVERSITY NWS, ND US (https://www.ncdc.noaa.gov/cdo-web/datasets/GHCND/stations/GHCND:USC00323621/detail). For ArboNET (CDC, 2022), we read the HTML and JavaScript to scrape the data. Figures were made with plotly (Sievert, 2020) available through https://plotly-r.com. All the statistical analyses were analyzed based on Stats and ggcorrplot (Kassambara, 2019) packages in R version 4.2.0 (R Core Team, 2022) available through https://www.R-project.org/ using GUI Rstudio 202207.1 Build 554 (RStudio Team, 2022) available through http://www.rstudio.com/. The map showing the spatial distribution of Culex species was visualized using GIS software QGIS 3.20.1 (QGIS.org, 2022) available at http://www.qgis.org. Other maps were delineated in R using sp package (Bivand et al., 2013; Pebesma & Bivand, 2005).
