# Supplementary material for: Temporal and Spatial Synchronicity in West Nile Virus Cases Along the Central Flyway, USA
Source: Geohealth. 2023 May 10;7(5):e2022GH000708. doi: 10.1029/2022GH000708 (PMC10171186; doi:10.1029/2022GH000708)
Supplement: Supplementary file 1 — Supporting Information S1 [file GH2-7-e2022GH000708-s001.pdf]

GeoHealth

Supporting Information for

Temporal and Spatial Synchronicity in West Nile virus Cases along the Central Flyway, USA

H. M. Hort[1], M. Ibaraki[2], and F. W. Schwartz[2]

[1]GSI Environmental Inc., Irvine, CA, USA

[2]School of Earth Sciences, The Ohio State University, Columbus, OH, USA

Corresponding author: Hiroko Hort (hmhort@gsienv.com)

Contents of this file

Figures S1 to S4

Introduction

Here we listed additional figures that support the main documentation.

Figure S1 illustrates the number of freeze days per year in Burleigh County. The freeze days were recorded from November to October. The greater the number, the colder the weather was in that year.

Figure S2 is the correlation of WNV cases in Burleigh County and the state of North Dakota. As demonstrated by Pearson's r correlation analysis. The results show that the WNV case numbers in Burleigh County are strongly correlated with the total number of disease cases in North Dakota ($r = 0.93$, $p < 0.001$). Please note that the data displayed in Figure S2 are log-transformed; as a result, years with zero WNV disease cases could not be plotted. For instance, Burleigh County had no disease cases in the year 2011. This information has been added in the figure caption.

Figure S3 shows the correlation analysis of total snowfall in Bismarck City and Grand Forks in North Dakota. Although the snowfall amounts were different, the annual trends in snowfall were similar between Bismarck and Grand Forks ($r = 0.69$, $p < 0.001$).

Figure S4 presents Pearson's r estimation of the annual West Nile virus data between Texas and the states along the northern Great Plains. This analysis was conducted using Pearson's r correlation analysis to address concerns about the limited data used for our statistical analysis. The sample numbers refer to the number of years used for the estimation of Pearson's r correlation analysis, starting from 2003. For example, "sample 2" means data from 2002 and 2003 were used. The trends become asymptotic starting from sample number 10.

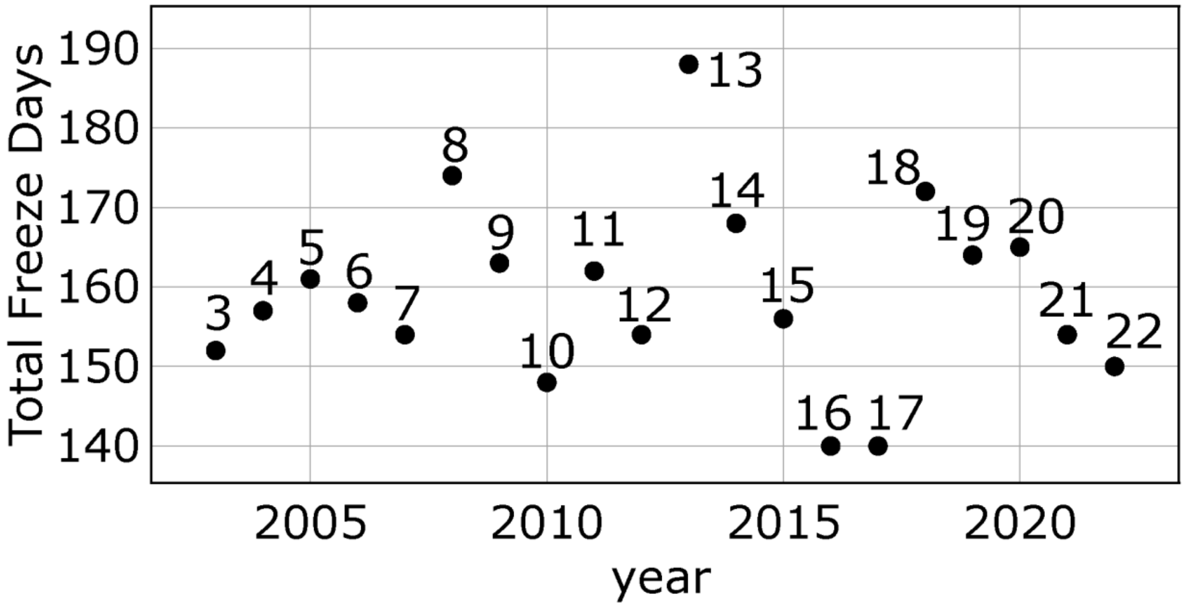

Figure S1. Annual freeze days in Burleigh County (e.g. 12 is 2012).

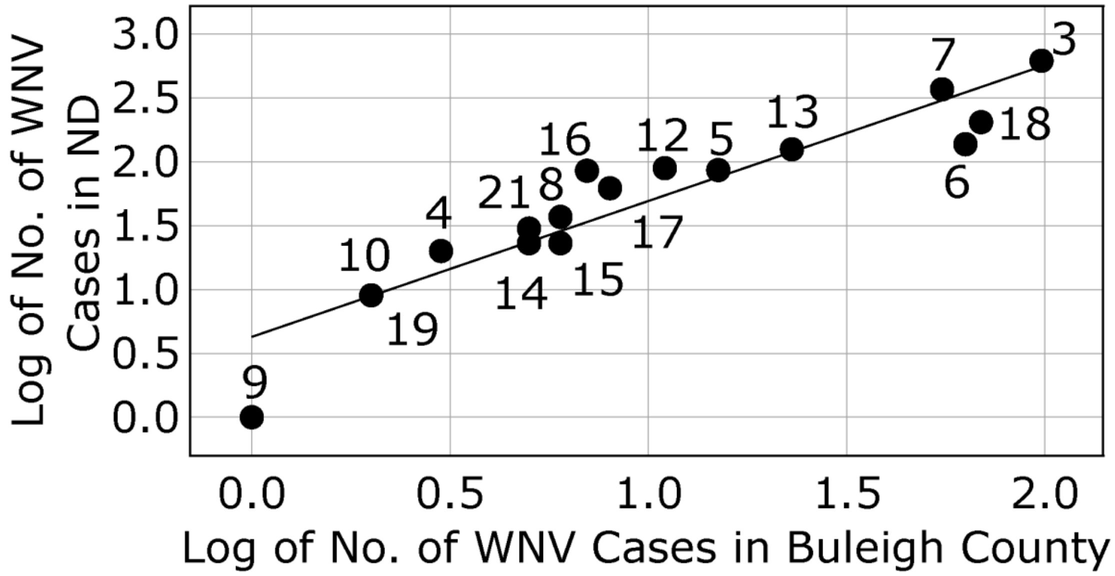

Figure S2. Correlation of annual WNV total WNV cases between Burleigh County in North Dakota and Statewide disease cases in North Dakota. The label is year (e.g. 12 is 2012). Burleigh county did not have any disease incidence in 2011.

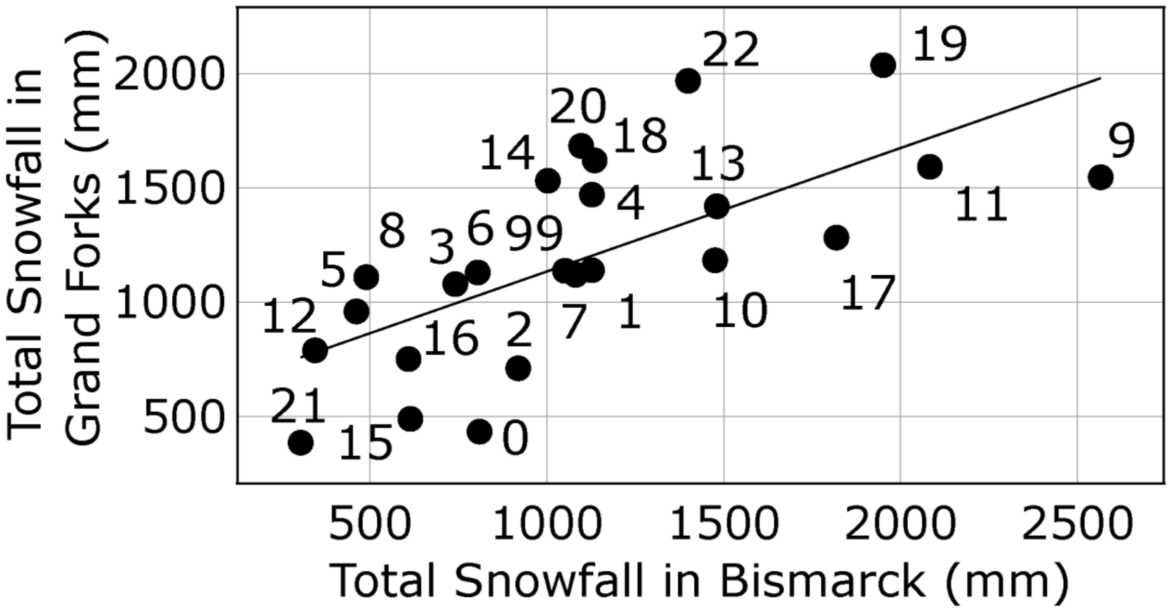

Figure S3. Correlation of total snowfall between Bismarck City in North Dakota and Grand Forks in North Dakota. Label is in year (e.g. 99 is 1999, 0 is 2000).

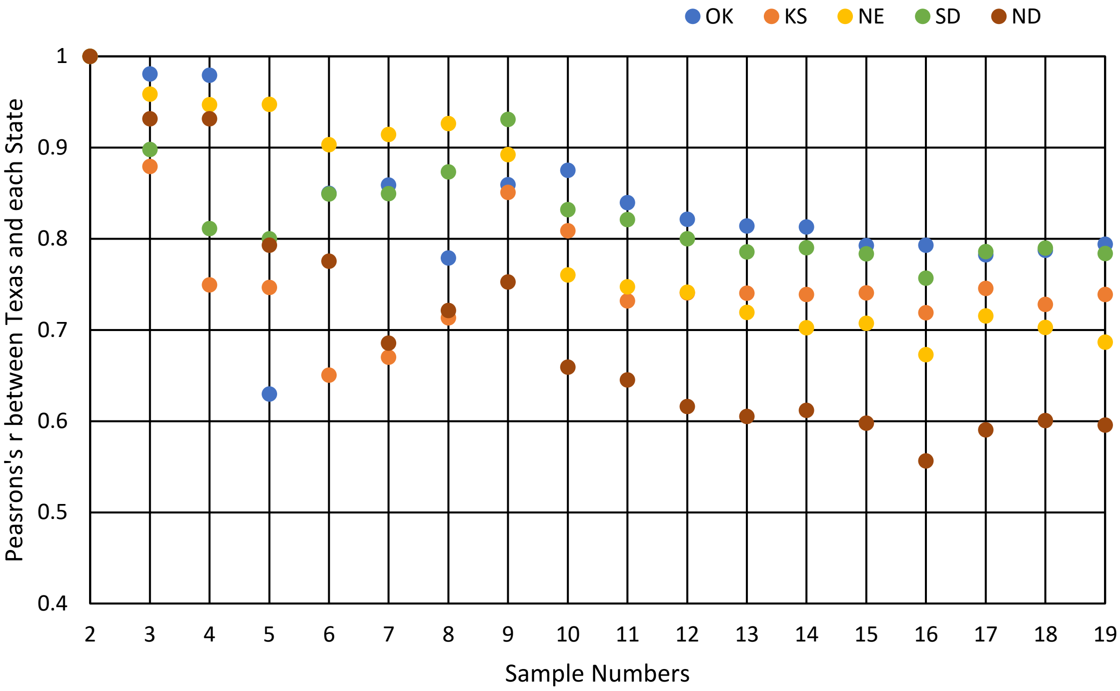

Figure S4. Statistical considerations on limited data in West Nile virus data.
